# Preliminary Research: Effectiveness of an Intervention Program Based on New Technologies for the Improvement of Cognitive and Motor Processes in Children and Adolescents with ADHD: A Randomized Controlled Trial

**DOI:** 10.3390/ejihpe15090167

**Published:** 2025-08-22

**Authors:** Berta Caro-Puértolas, Inmaculada Báez-Tavero, Laura Lemus-Corchero, Laura Rodríguez-Ruiz, Celia Esther Cerezo-Casillas, Ana Inés Cosa-Aguirre, María Dolores Apolo-Arenas, Alejandro Caña-Pino

**Affiliations:** 1 Department of Medical-Surgical Therapy, Medicine and Health Sciences Faculty, Extremadura University, 06006 Badajoz, Spain; bertacaro@unex.es (B.C.-P.); alejandrocp@unex.es (A.C.-P.); 2 Research Group PhysioH (Fisioterapia e Hipoterapia), University of Extremadura, 06006 Badajoz, Spain; 3 Thercli Clinical Therapy Center, 06400 Don Benito, Spain

**Keywords:** Attention Deficit Hyperactivity Disorder, ADHD, cognitive training, neurocognitive therapy, active video games, Nintendo Switch, processing speed

## Abstract

Attention Deficit Hyperactivity Disorder (ADHD) is one of the most prevalent neurodevelopmental disorders in childhood and adolescence, characterized by symptoms of inattention, hyperactivity, and impulsivity. These symptoms often interfere with academic, social, and family functioning. In recent years, the use of digital tools and video games has garnered attention as an innovative and engaging approach for neurocognitive rehabilitation. The primary objective of this randomized controlled study was to investigate the comparative effects of two cognitive intervention approaches—one based on new technologies and one using traditional methods—on attention, inhibitory control, and processing speed in children and adolescents diagnosed with ADHD. Thirty-three participants aged 6–17 years were randomly assigned to either an experimental group (n = 17), which received Nintendo Switch-based therapy, or a control group (n = 16), which received traditional board game therapy. Both interventions lasted 8 weeks and included 16 sessions. Outcomes were assessed using the WISC-V, STROOP, and CARAS-R tests. Results showed significant within-group improvements in both groups. The control group exhibited gains in sustained attention and inhibitory control (CARAS-R and STROOP tests, *p* < 0.05), while the experimental group improved significantly in processing speed, as measured by the WISC-V (*p* = 0.001). However, no significant differences were found between groups. These findings suggest that both interventions may be effective for enhancing different cognitive processes in children with ADHD. Importantly, the use of familiar digital technologies like the Nintendo Switch may promote greater motivation and adherence to treatment. Further research with larger samples and long-term follow-up is warranted to validate and extend these preliminary findings, as the current sample size was not powered to detect medium or small effects.

## 1. Introduction

Attention Deficit Hyperactivity Disorder (ADHD) is one of the most prevalent disorders in childhood and adolescence in recent years (Rusca-Jordán & Cortez Vergara, 2020). According to the Diagnostic and Statistical Manual of Mental Disorders, Fifth Edition (DSM-5), ADHD is classified as a neurodevelopmental disorder characterized primarily by inattention and hyperactivity/impulsivity symptoms (American Psychiatric Association, 2013). In addition to this general symptomatology, it causes clinically significant distress and interferes with functioning across family, school, and social domains (Llanos Lizcano et al., 2019).

These cognitive dysfunctions are especially evident in daily activities that demand attention, working memory, and behavioral regulation. Executive function impairments in ADHD typically include difficulties in inhibitory control, cognitive flexibility, and sustained attention (Muszynska & Ossmy, 2025). Emotional dysregulation in this population often presents as poor frustration tolerance, sudden mood shifts, and challenges in modulating emotional intensity and expression—factors that further impact functioning across home, school, and peer settings (Carrasco-Chaparro, 2022; Song et al., 2025; Sordo et al., 2021). These deficits significantly interfere with social behavior, academic performance, and adaptive functioning (Song et al., 2025).

Although pharmacological treatments, such as methylphenidate, are effective for reducing core symptoms in 60–75% of patients, they often require complementary non-pharmacological strategies to target cognitive deficits and enhance functional outcomes (García Ron et al., 2015). While these medications can effectively reduce hyperactivity and improve attention in many patients, they may cause side effects such as appetite suppression, sleep disturbances, or mood alterations. Moreover, pharmacological treatment alone does not address executive functioning deficits or social–emotional skills, which are critical for adaptive functioning. These limitations support the need for multimodal approaches combining behavioral and cognitive therapies. As such, clinical guidelines advocate for multimodal interventions that combine medication with behavioral and cognitive therapies.

Among the most evidence-based non-pharmacological interventions are behavioral parent training, classroom management strategies, peer-mediated interventions, and cognitive programs aimed at improving planning, attention, and organizational skills (Pareja & Muñoz, 2022). These therapies are widely recommended as first-line or adjunctive treatments in clinical practice.

Clinical practice guidelines and evidence-based protocols for ADHD recommend the use of both pharmacological and psychological interventions (Johnston & Park, 2015; Macia, 2012; Wolraich et al., 2019).

These data highlight the importance of the use of conventional therapies for symptom improvement with children diagnosed with ADHD, and moreover, previous studies suggest that the use of video games in designed and controlled interventions promote optimal cognitive performance, as they provide continuous feedback and improve attention and inhibitory control (Granic et al., 2013; Tahiroglu et al., 2010). In recent years, serious games—digital applications specifically designed for therapeutic, educational, or cognitive enhancement purposes—have gained growing recognition in the field of neurorehabilitation. These games integrate clinical principles into engaging, interactive environments that support executive function training and attentional control. Evidence suggests that serious games can enhance cognitive engagement through real-time feedback, task repetition, and motivational elements (Lumsden et al., 2016; Primack et al., 2012). While most clinical research has focused on custom-developed applications or exergaming platforms like Xbox Kinect, less is known about the cognitive benefits of commercial gaming systems. Commercial games not originally designed for therapeutic use may still possess characteristics conducive to cognitive development, including task-switching, problem-solving, and inhibitory control demands—features relevant for ADHD populations (Granic et al., 2013). However, most studies focus on platforms such as Xbox Kinect or custom-designed applications. There is a lack of research on commercially available consoles like the Nintendo Switch, particularly regarding their cognitive impact in ADHD populations. Various tools are employed to assess cognitive and behavioral features of ADHD, in addition to general neuropsychological instruments like the Wechsler Intelligence Scale for Children, Fifth Edition (WISC-V), and the CARAS-R test for attention. More specific tools such as the Conners 3rd Edition (Conners 3) and the ADHD Rating Scale-5 are commonly used to evaluate symptom severity and functional impact in clinical and educational settings (Malhotra et al., 2025).

This gap motivates the exploration of whether widely accessible entertainment technologies can be repurposed for therapeutic use and yield comparable cognitive benefits.

The primary objective of this randomized controlled study was to investigate the comparative effects of two cognitive intervention approaches—one based on new technologies and one using traditional methods—on attention, inhibitory control, and processing speed in children and adolescents diagnosed with ADHD.

## 2. Materials and Methods

### 2.1. Study Design

The study was conducted in accordance with CONSORT recommendations, following the Helsinki declaration and approved by the Bioethics and Biosafety committee of the University of Extremadura (registration number: 155//2023). In addition, it was registered on clinicaltrials.gov with registration number NCT06477575. All participants and their legal guardians agreed to the study voluntarily. Written informed consent was obtained from the parents or legal representatives of all minors, in accordance with ethical research standards involving children and adolescents.

A randomized clinical trial was conducted with a total of 33 children and adolescents aged 6 to 17 years, all diagnosed with ADHD and recruited from the Thercli Center in Don Benito (Badajoz, Spain). Participants had a confirmed clinical diagnosis of ADHD, were free of comorbid neurological or psychiatric conditions, and were not receiving psychopharmacological treatments unrelated to ADHD. All participants had been diagnosed with ADHD at least six months before enrollment and were undergoing stable pharmacological treatment—primarily with methylphenidate or lisdexamfetamine—for a minimum of three months. No participants were engaged in additional psychological or cognitive–behavioral therapies during the study period, ensuring uniformity in intervention exposure.

**Figure 1:**
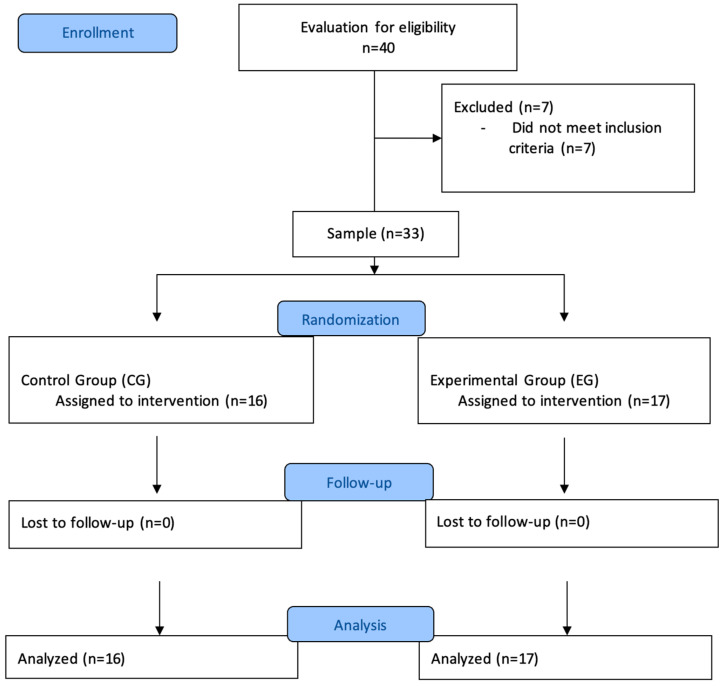
CONSORT flowchart.

The sample was randomly allocated into two groups using a simple even–odd assignment: experimental group (EG): composed of 17 patients who received neurocognitive therapy based on new technologies, and control group (CG): composed of 16 patients who received conventional neurocognitive therapy for the treatment of ADHD (Figure 1). The allocation was simple, using even numbers for the EG and odd numbers for the CG. Due to the nature of the study, blinding was not possible. The treatment protocol was as follows: Participants in the EG received the cognitive neurorehabilitation program based on new technologies. The Nintendo Switch console was used for this purpose. For the CG, a conventional cognitive neurorehabilitation program was developed based on the use of traditional games and a chip book. The Nintendo Switch is a hybrid video game console developed by Nintendo, featuring both handheld and motion-based interactive capabilities. It supports a range of cognitively engaging games and provides real-time feedback, making it a promising tool for neurocognitive stimulation. Its popularity among children and adolescents also increases engagement and adherence during therapeutic sessions. Neurocognitive therapy, in this context, refers to structured interventions aimed at improving cognitive processes such as attention, inhibitory control, and processing speed through targeted tasks and feedback. Each program followed a semi-structured weekly plan adapted for pediatric populations and designed to progressively increase cognitive demand. The sessions included activities focused on selective attention, cognitive flexibility, and response inhibition. The interventions were delivered by a multidisciplinary team composed of one licensed neurophysiologist and two trained cognitive therapists, all with over five years of experience in pediatric neurodevelopmental intervention. Each session was therapist-guided to ensure adherence to cognitive objectives and to adjust task difficulty based on the individual needs of each participant.

Both therapeutic intervention programs were chosen because they work on selective attention, concentration, and processing speed, the variables under study. The Nintendo Switch intervention included games such as “Brain Training” and “Big Brain Academy,” which are designed to stimulate working memory, attention span, and processing speed through interactive mini-challenges. Each session was structured to progressively increase task complexity, providing immediate feedback to enhance motivation and engagement. Therapists guided gameplay to ensure that the cognitive targets of the intervention were met.

Both groups had 2 sessions per week for 8 weeks, each consisting of 30 min of effective therapy of therapy each, for a total of 16 therapy sessions (Table 1). The initial assessment was carried out in December 2023 and the therapy sessions and final assessment during the months of January to March 2024.

This table outlines the structured progression of cognitive targets and tasks across the 8-week intervention period for both groups. The experimental group used Nintendo Switch games (e.g., Brain Training, Big Brain Academy) designed to enhance attention, processing speed, and inhibitory control through interactive challenges. The control group followed a parallel structure using traditional cognitive board games and paper-based exercises aligned with the same therapeutic objectives. All sessions were therapist-guided and adapted to participant performance.

### 2.2. Variables/Instruments

- *Wechsler Intelligence Scale for Children—Fifth Edition (WISC-V):* This standardized test assesses a range of cognitive functions in children aged 6–16 years. For this study, we used the Processing Speed Index, which evaluates visual processing and graphomotor speed. The WISC-V has excellent psychometric properties, with internal consistency coefficients ranging from 0.88 to 0.96 and strong construct validity (Wechsler, 2015).
- *Stroop Color and Word Test:* The STROOP test measures cognitive interference and inhibitory control by evaluating the subject’s ability to inhibit a prepotent response (e.g., reading a word) in favor of a conflicting one (e.g., naming a color). It has demonstrated good reliability (test–retest coefficients around 0.80) and is widely used in clinical neuropsychology (Stroop, 1935).
- *CARAS-R (Test of Perception of Similarities and Differences):* CARAS-R assesses sustained and selective attention through rapid identification of matching or non-matching schematic faces. It evaluates both accuracy and impulsivity via error and success rates. The instrument shows acceptable internal consistency (α = 0.78–0.84) and strong applicability in pediatric populations (Thurstone & Yela, 2012).

### 2.3. Statistical Analysis

Data analysis was performed with the Jamovi (2020) project program. A descriptive analysis was performed for each of the variables. The normality of the variables was assessed using the Shapiro–Wilk test, which showed a normal distribution for all variables (except for the compulsivity index), and therefore, parametric tests were adequate for the rest of the variables. Independent and related sample *t*-tests were used for between-group and within-group comparisons, respectively. Nonparametric tests (Wilcoxon and Mann–Whitney) were applied when strong skewness was observed (compulsivity index variable). Data were reported as mean and SD. A 2-way repeated-measures analysis of variance (ANOVA) was performed to analyze the interaction effects of time [at baseline (pre-treatment), after treatment (post-treatment)] in the 2 groups. These results were analyzed separately by repeated-measures analysis of variance (with Greenhouse–Geisser sphericity correction) to compare the groups. The effect size for within-group mean differences was calculated using Cohen’s d coefficient, with d = 0.2 being considered a “small” effect size, 0.5 a “medium” effect size, and 0.8 a “large” effect size. A significance level of *p* < 0.05 was used.

## 3. Results

This study included 33 children diagnosed with ADHD, of whom 17 belonged to the EG, with a mean age of 10.7 years, and 16 belonged to the CG, with a mean age of 9.06 years. The percentage by sex of the total sample was 69.7% boys and 30.3% girls.

Table 2 includes the pre- and post-treatment values of the numerical variables compulsivity index, interference, and WISC-V, as well as the data obtained for the variables related to the interpretations of these variables (CARAS, STROOP, and WISC-V interpretations) and the differences between the measures before and after the intragroup treatment.

In the control group, within-group analyses showed statistically significant differences before and after the intervention for the variable CARAS interpretation (*p* = 0.03) and the variable interference measured with the STROOP test (*p* = 0.02).

**Figure 2:**
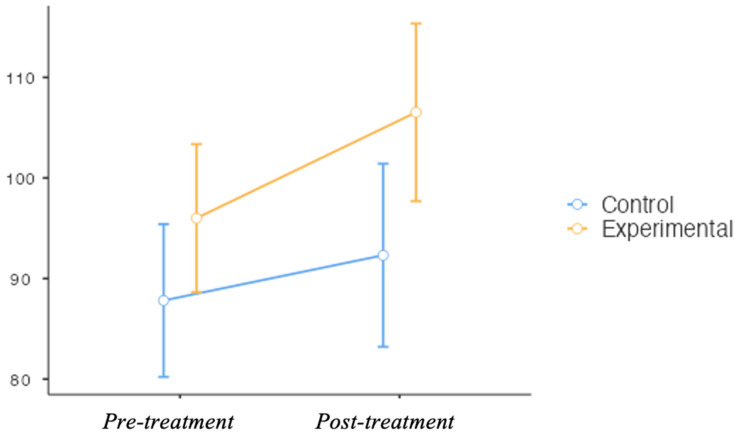
Intergroup comparison of processing speed index scores (WISC-V) before and after the intervention. The graph displays mean scores and standard deviations for the experimental group (Nintendo Switch) and control group (traditional therapy). Although no statistically significant differences were found between groups, descriptive trends suggest improvements in both conditions.

The experimental group showed statistically significant differences before and after the intervention for the WISC-V test (*p* = 0.001) and its interpretation (*p* = 0.02) (*p* < 0.05) (Figure 2).

In the intergroup analysis (Table 3), no statistically significant differences were observed between groups or for the time × group interaction in any of the variables studied (all *p* > 0.05). Although the between-group comparisons did not yield significant results, some descriptive shifts were noted within groups. For example, in the experimental group, participants transitioned from a “low inhibition” profile to “within normal limits” on the STROOP interpretation scale. However, these observations should be interpreted with caution, as they are not supported by statistically significant findings.

## 4. Discussion

This study evaluated whether a cognitive intervention using a commercial video game platform (Nintendo Switch) could improve executive functioning in children and adolescents with ADHD compared to a traditional board game approach. Although no statistically significant differences were observed between groups, both interventions showed clinically relevant within-group improvements in distinct cognitive domains. The traditional format appeared to support sustained attention and inhibitory control, while the digital format showed descriptive gains in processing speed. These modality-specific effects align with previous evidence suggesting that the sensory and structural demands of different activities may engage executive functions in diverse ways (Cortese et al., 2015).

These findings are consistent with earlier studies examining the efficacy of cognitive training using video games. For example, Benzing and Schmidt (2019) reported improvements in inhibitory control following Kinect-based interventions. In contrast, Weerdmeester et al. (2016) did not observe such effects, underscoring the variability of outcomes depending on platform type and protocol design. Our results extend this literature by exploring the Nintendo Switch as a tool for neurocognitive therapy—an accessible and commercially available option that has not been extensively studied in ADHD populations.

Participants in our study continued their pharmacological treatment at a stable dose throughout the intervention, in accordance with guidelines recommending combined pharmacological and non-pharmacological management for ADHD (Johnston & Park, 2015; Wolraich et al., 2019). The observed trends may reflect a complementary effect of stimulant medication and cognitive training, although further controlled studies are needed to disentangle their contributions.

Although the STROOP test showed descriptive improvements in the experimental group, these changes did not reach statistical significance and should be interpreted cautiously. One possible explanation for the lack of intergroup effects lies in the type of video game used. While exergaming systems such as Xbox Kinect incorporate physical activity, our Nintendo Switch-based protocol focused on fine motor and cognitive skills, which may preferentially activate specific executive domains. The relatively short intervention period (16 sessions) may also have limited cognitive adaptation.

In our control group, significant improvements in processing speed were detected through the WISC-V, consistent with findings by Bikic et al. (2018) after 24 weeks of computerized cognitive training, and Rosa et al. (2017), who observed similar gains following 48 sessions of computer-guided therapy. Likewise, improvements in sustained attention and inhibitory control among participants receiving conventional neurocognitive therapy echo results from prior studies using traditional interventions (Bikic et al., 2018; Bruce et al., 2017; Dovis et al., 2015; Johnstone et al., 2017; Mishra et al., 2016; Rosa et al., 2017; Simone et al., 2018; Weerdmeester et al., 2016). Some studies (e.g., Johnstone et al., 2017; Weerdmeester et al., 2016) also explored whether video games could reduce ADHD symptoms, while Faraone et al. (2016) investigated the diagnostic potential of such technologies in populations aged 6–17 years. The duration of these longitudinal studies ranged from 6 to 25 sessions, reinforcing the importance of protocol length in evaluating cognitive outcomes.

Taken together, these preliminary results suggest modality-specific trends that warrant further exploration with larger samples and extended protocols. This study contributes to the literature by demonstrating the feasibility of using familiar, motivating, and low-cost technologies in structured cognitive interventions for ADHD.

### 4.1. Practical and Clinical Implications

- High Motivation and Engagement: The use of commercially available gaming technologies like the Nintendo Switch may significantly enhance adherence to neurocognitive therapy in children and adolescents with ADHD due to their strong motivational appeal.
- Complementary Tool: Although no statistically significant differences were found between interventions, observed trends suggest that video game-based therapy can complement traditional approaches by enhancing processing speed, while conventional therapies may be more effective for attention and inhibitory control.
- Group-Based Delivery: Implementing these interventions in group settings may further increase motivation, social engagement, and cost-effectiveness—especially important in clinical or educational contexts with limited resources.
- Accessibility and Scalability: The Nintendo Switch platform offers a low-cost, scalable option for widespread implementation in schools, clinics, or home-based programs, making it particularly suitable for under-resourced environments.
- Clinical Integration: These findings support the integration of gamified digital tools into multimodal ADHD treatment plans as a means of increasing participation, maintaining engagement, and targeting specific cognitive domains.

### 4.2. Limitations and Future Lines

This study presents several limitations that should be acknowledged. First, the small sample size restricts the statistical power and generalizability of the findings. Although the study employed a randomized design, a formal a priori power analysis was not conducted. Post hoc estimation suggests that detecting medium effect sizes (Cohen’s d ≈ 0.5) with 80% power at α = 0.05 would require approximately 64 participants (32 per group). Thus, the current sample may have lacked the statistical sensitivity to identify subtle between-group differences. Second, the absence of long-term follow-up limits our understanding of the durability of the observed cognitive changes. Third, without a passive control group, it is difficult to disentangle specific treatment effects from natural progression or placebo influences. Additionally, motivational bias due to the appeal of the Nintendo Switch may have impacted engagement levels. Lastly, as participants were drawn from a single clinical site, the findings may not extend to broader or more heterogeneous ADHD populations. Future research should address these issues through larger, multicenter trials with extended follow-up periods and rigorous control conditions.

Future studies should also consider comparing commercially available video games with clinically validated platforms such as EndeavorRx^®^, which has demonstrated efficacy in improving attention in children with ADHD. Evaluation of cost-effectiveness, user satisfaction, and implementation feasibility in diverse real-world contexts will also be essential to optimizing the therapeutic use of digital interventions.

## 5. Conclusions

Although no statistically significant differences were observed between the two modalities, both interventions yielded clinically relevant improvements in distinct executive functions. These findings offer initial support for integrating technology-based platforms, such as the Nintendo Switch, into multimodal ADHD treatment. Importantly, the use of familiar, engaging tools could enhance adherence and scalability in real-world settings. Further large-scale studies with long-term follow-up are needed to confirm these trends.

## Figures and Tables

**Table 1:** Weekly objectives and core activities of the cognitive intervention programs.

Week	Objective	Experimental Group (Nintendo Switch)	Control Group (Traditional Games)
1	Baseline assessment, familiarization	“Brain Training” warm-up, guided interface use	Visual attention puzzles, basic instruction games
2	Focused attention	“Big Brain Academy” timed tasks	Matching card games, rule-based sorting tasks
3	Processing speed	Mini-games with increasing time constraints	Timed maze games, shape sequencing
4	Inhibitory control	Tasks with distractors and delayed responses	Simon says, “Don’t clap,” impulse-control game
5	Working memory	Number and object recall challenges	Board games with sequence memorization
6	Cognitive flexibility	Task-switching games (e.g., color vs. shape rules)	Color–shape card sorting with rule changes
7	Integration of skills	Mixed-modality challenges, feedback-based scoring	Combined rule puzzles, cooperative logic games
8	Final review and consolidation	Replay of prior challenges, therapist feedback	Repetition of previous games, strategy reflection

**Table 2:** Intragroup analysis.

	Control Group (n = 16) Experimental Group (n = 17)	Pre-Treatment Mean ± SD (Score)	Post-Treatment Mean ± SD (Score)	Intragroup Mean Difference (Δ) with 95% Confidence Interval (CI)	Pre-Post Treatment Effect Size (d)	Pre-Post Treatment *p* Value
Compulsivity index (CARAS test)	Control	82.8 ± 16.8	89.9 ± 20.8	10.4 (−20.5, 0.75)	−0.343	0.06 ^ττ^
	Experimental	90.4 ± 10.3	90.8 ± 16.2	2.25 (−12.5, 7.65)	−0.12	0.71 ^ττ^
CARAS interpretation	Control	2.06 ± 0.68	2.44 ± 0.73	1.00 (1.00, 1.00)	−1.00	0.03 ^τ^*
	Experimental	1.88 ± 0.49	2.00 ± 0.61	4.31 (−1.00, 1.98)	−0.33	0.43 ^τ^
Interference (STROOP test)	Control	1.19 ± 6.55	6.02 ± 6.51	6.33 (−9.26, −0.27)	−0.60	0.02 ^τ^*
	Experimental	0.35 ± 6.31	1.99 ± 13.4	2.17 (−7.17, 3.66)	−0.28	0.63 ^τ^
STROOP interpretation	Control	1.19 ± 6.55	2.19 ± 0.66	1.00 (−1.00, −6.72)	−0.43	0.27 ^τ^
	Experimental	0.35 ± 6.31	2.00 ± 0.79	1.93 (−1.00, 1.00)	0.20	0.58 ^τ^
WISC-V TEST	Control	87.8 ± 15.4	92.3 ± 19.4	7.00 (−11.50, 3.00)	−0.35	0.17 ^τ^
	Experimental	96.0 ± 14.3	107 ± 16.3	12.00 (−17.00, −7.00)	−0.98	0.001 ^τ^*
WISC-V interpretation	Control	3.06 ± 1.34	3.50 ± 1.32	1.00 (−1.50, 0.5)	−0.53	0.11 ^τ^
	Experimental	3.76 ± 1.15	4.41 ± 1.06	1.00 (−1.50, −1.00)	−0.71	0.02 ^τ^*

^τ^: *T*-test for paired samples; ^ττ^: Wilcoxon test; SD: standard deviation; CI: confidence interval; d: Cohen’s d; control group: conventional neurocognitive therapy; experimental group: neurocognitive therapy using new technologies; * *p* < 0.05: statistical significance; Δ: mean score difference.

**Table 3:** Intergroup analysis.

	Intergroup Mean Difference (Δ) with 95% Confidence Interval (CI)	Pre-Post Treatment *p* Value
Compulsivity index (CARAS Test)	−6.06 (−16.5, 2.30)	0.18 ^ττ^
CARAS interpretation	−0.26 (−0.69, 0.18)	0.24 ^τ^
Interference (STROOP test)	−3.52 (−8.73, 3.28)	0.41 ^τ^
STROOP interpretation	−0.31 (−0.85, 0.24)	0.26 ^τ^
WISC-V TEST	5.00 (−3.00, 12.00)	0.11 ^τ^
WISC-V interpretation	0.21 (−0.51, 0.93)	0.56 ^τ^

^τ^: Repeated measures ANOVA ^ττ^: Mann–Whitney test; CI: confidence interval; control group: conventional neurocognitive therapy; experimental group: neurocognitive therapy using new technologies; Δ: mean score difference.

## Data Availability

The data supporting the findings of this study are not publicly available due to ethical restrictions and participant confidentiality, in accordance with the approval granted by the University of Extremadura’s Bioethics and Biosafety Committee. Data may be made available from the corresponding author upon reasonable request and with appropriate institutional and ethical approvals.

## Abbreviations

The following abbreviations are used in this manuscript:

ADHD	Attention Deficit Hyperactivity Disorder
WISC-V	Wechsler Intelligence Scale for Children, Fifth Edition
CARAS	Test of Perception of Similarities and Differences
STROOP	Stroop Color and Word Test
EG	Experimental Group
CG	Control Group
RCT	Randomized Controlled Trial
IRB	Institutional Review Board

## References

[B1-ejihpe-15-00167] American Psychiatric Association (2013). Diagnostic and statistical manual of mental disorders.

[B2-ejihpe-15-00167] Benzing V., Schmidt M. (2019). The effect of exergaming on executive functions in children with ADHD: A randomized clinical trial. Scandinavian Journal of Medicine & Science in Sports.

[B3-ejihpe-15-00167] Bikic A., Leckman J. F., Christensen T., Bilenberg N., Dalsgaard S. (2018). Attention and executive functions computer training for attention-deficit/hyperactivity disorder (ADHD): Results from a randomized, controlled trial. European Child & Adolescent Psychiatry.

[B4-ejihpe-15-00167] Bruce C. R., Unsworth C. A., Dillon M. P., Tay R., Falkmer T., Bird P., Carey L. M. (2017). Hazard perception skills of young drivers with Attention Deficit Hyperactivity Disorder (ADHD) can be improved with computer based driver training: An exploratory randomised controlled trial. Accident; Analysis and Prevention.

[B5-ejihpe-15-00167] Carrasco-Chaparro X. (2022). On Attention Deficit Hyperactivity Disorder: Consolidations, updates and perspectives. Revista Medica Clinica Las Condes.

[B6-ejihpe-15-00167] Cortese S., Ferrin M., Brandeis D., Buitelaar J., Daley D., Dittmann R. W., Holtmann M., Santosh P., Stevenson J., Stringaris A., Zuddas A., Sonuga-Barke E. J. S. (2015). Cognitive training for attention-deficit/hyperactivity disorder: Meta-analysis of clinical and neuropsychological outcomes from randomized controlled trials. Journal of the American Academy of Child and Adolescent Psychiatry.

[B7-ejihpe-15-00167] Dovis S., Van Der Oord S., Wiers R. W., Prins P. J. M. (2015). Improving executive functioning in children with ADHD: Training multiple executive functions within the context of a computer game. A randomized double-blind placebo controlled trial. PLoS ONE.

[B8-ejihpe-15-00167] Faraone S. V., Newcorn J. H., Antshel K. M., Adler L., Roots K., Heller M. (2016). The groundskeeper gaming platform as a diagnostic tool for attention-deficit/hyperactivity disorder: Sensitivity, specificity, and relation to other measures. Journal of Child and Adolescent Psychopharmacology.

[B9-ejihpe-15-00167] García Ron A., Blasco Fontecilla H., Huete Hernani B., Sabaté J. (2015). Tratamiento farmacológico estimulante del TDAH. Revista Española de Pediatría: Clínica e Investigación.

[B10-ejihpe-15-00167] Granic I., Lobel A., Engels R. C. M. E. (2013). The benefits of playing video games. American Psychologist.

[B11-ejihpe-15-00167] Johnston C., Park J. L. (2015). Interventions for attention-deficit hyperactivity disorder: A year in review. Current Developmental Disorders Reports.

[B12-ejihpe-15-00167] Johnstone S. J., Roodenrys S. J., Johnson K., Bonfield R., Bennett S. J. (2017). Game-based combined cognitive and neurofeedback training using Focus Pocus reduces symptom severity in children with diagnosed AD/HD and subclinical AD/HD. International Journal of Psychophysiology: Official Journal of the International Organization of Psychophysiology.

[B13-ejihpe-15-00167] Llanos Lizcano L. J., García Ruiz D. J., González Torres H. J., Puentes Rozo P. (2019). Trastorno por déficit de atención e hiperactividad (TDAH) en niños escolarizados de 6 a 17 años. Pediatría Atención Primaria.

[B14-ejihpe-15-00167] Lumsden J., Edwards E. A., Lawrence N. S., Coyle D., Munafò M. R. (2016). Gamification of cognitive assessment and cognitive training: A systematic review of applications and efficacy. JMIR Serious Games.

[B15-ejihpe-15-00167] Macia D. (2012). TDAH en la infancia y la adolescencia: Concepto, evaluación y tratamiento.

[B16-ejihpe-15-00167] Malhotra S., Kishore M. T., De Sousa A. (2025). Clinical practice guidelines on cognitive impairment and ADHD—Assessment and management. Indian Journal of Psychiatry.

[B17-ejihpe-15-00167] Mishra J., Sagar R., Joseph A. A., Gazzaley A., Merzenich M. M. (2016). Training sensory signal-to-noise resolution in children with ADHD in a global mental health setting. Translational Psychiatry.

[B18-ejihpe-15-00167] Muszynska M., Ossmy O. (2025). A multivariate analysis reveals benefits of specific ADHD characteristics in trial-and-error learning. PsyArXiv.

[B19-ejihpe-15-00167] Pareja M. Á. V., Muñoz M. d. l. F. R. (2022). Manual de terapia de conducta en la infancia.

[B20-ejihpe-15-00167] Primack B. A., Carroll M. V., McNamara M., Klem M. L., King B., Rich M., Chan C. W., Nayak S. (2012). Role of video games in improving health-related outcomes. American Journal of Preventive Medicine.

[B21-ejihpe-15-00167] Rosa V. d. O., Schmitz M., Moreira-Maia C. R., Wagner F., Londero I., Bassotto C. d. F., Moritz G., de Souza C. d. S., Rohde L. A. P. (2017). Computerized cognitive training in children and adolescents with attention deficit/hyperactivity disorder as add-on treatment to stimulants: Feasibility study and protocol description. Trends in Psychiatry and Psychotherapy.

[B22-ejihpe-15-00167] Rusca-Jordán F., Cortez Vergara C. (2020). Trastorno por déficit de atención con hiperactividad (TDAH) en niños y adolescentes. Una revisión clínica. Revista de Neuro-Psiquiatría: (RNP).

[B23-ejihpe-15-00167] Simone M., Viterbo R. G., Margari L., Iaffaldano P. (2018). Computer-assisted rehabilitation of attention in pediatric multiple sclerosis and ADHD patients: A pilot trial. BMC Neurology.

[B24-ejihpe-15-00167] Song X., Hou Y., Shi W., Wang Y., Fan F., Hong L. (2025). Exploring the impact of different types of exercise on working memory in children with ADHD: A network meta-analysis. Frontiers in Psychology.

[B25-ejihpe-15-00167] Sordo S. Á., Cantero-García M., Garrido-Hernansaiz H., Sánchez-Iglesias I., Mas J. S. (2021). Atención sostenida y selectiva en subtipos de TDAH y en trastorno de aprendizaje: Una comparación clínica. Electronic Journal of Research in Education Psychology.

[B26-ejihpe-15-00167] Stroop J. R. (1935). Studies of interference in serial verbal reactions. Journal of Experimental Psychology.

[B27-ejihpe-15-00167] Tahiroglu A. Y., Celik G. G., Avci A., Seydaoglu G., Uzel M., Altunbas H. (2010). Short-term effects of playing computer games on attention. Journal of Attention Disorders.

[B28-ejihpe-15-00167] Thurstone L. L., Yela M. (2012). Caras-R. Test de percepción de test de percepción de diferencias—Revisado.

[B29-ejihpe-15-00167] Wechsler D. (2015). Wechsler intelligence scale for children—Fifth edition (WISC–V).

[B30-ejihpe-15-00167] Weerdmeester J., Cima M., Granic I., Hashemian Y., Gotsis M. (2016). A feasibility study on the effectiveness of a full-body videogame intervention for decreasing Attention Deficit Hyperactivity Disorder symptoms. Games for Health Journal.

[B31-ejihpe-15-00167] Wolraich M. L., Hagan J. F., Allan C., Chan E., Davison D., Earls M., Evans S. W., Flinn S. K., Froehlich T., Frost J., Holbrook J. R., Lehmann C. U., Lessin H. R., Okechukwu K., Pierce K. L., Winner J. D., Zurhellen W. (2019). Clinical practice guideline for the diagnosis, evaluation, and treatment of attention-deficit/hyperactivity disorder in children and adolescents. Pediatrics.

